# New frontiers in the neuropsychopharmacology of mental illness

**DOI:** 10.3389/fphar.2014.00212

**Published:** 2014-09-17

**Authors:** Thibault Renoir

**Affiliations:** ^1^Behavioural Neuroscience, Florey Institute of Neuroscience and Mental Health, University of MelbourneMelbourne, VIC, Australia; ^2^Faculty of Medicine, Dentistry and Health Sciences, University of MelbourneMelbourne, VIC, Australia

**Keywords:** anxiety/depression/mood disorders, schizophrenia, addiction, gene × environment (G × E) interaction, serotonin, glutamate/GABA system

This Research Topic aims to cover recent progress in research studying how genetic make-up and environmental factors can contribute to the development of mental disorders such as anxiety, depression, schizophrenia, and psychoactive substances abuse. It has brought together leading experts in the field to address these questions from different angles in eleven reviews, seven original research articles and two theoretic/opinion papers.

The first three articles describe several techniques which are valuable tools to study the role of neurotransmitters such as serotonin (5-HT) in the pathophysiology and the treatment of psychiatric disorders. First, Prof. Gardier (2013) nicely summarizes the main advantages as well as some limitations of using microdialysis in wildtype (WT) and knockout (KO) mice. His team showed that paroxetine-induced increased in cortical 5-HT extracellular level was enhanced in 5-HT_1A_ receptor KO mice compared to WT animals. Then, by performing loose-seal cell-attached electrophysiological recordings in 5-HT transporter knockout (Sert^−/−^) and tryptophan hydroxylase-2 knockout (Tph2^−/−^) mice, Araragi et al. (2013) demonstrate that the sensitivity of somatodendritic 5-HT_1A_ receptors does not predict the magnitude of 5-HT neuron auto-inhibition. Finally, Mendez-David et al.'s (2013) results suggest that isolation of peripheral blood mononuclear cells (PBMCs) from mice by submandibular bleeding is a useful technique to screen putative biomarkers relevant to the pathophysiology of mood disorders such as β-arrestin 1. They found that the reduced β-arrestin 1 levels found in PBMCs from anxious/depressed mice was restored to normal levels following chronic treatment with fluoxetine.

The following eleven articles provide excellent insights into the interaction between gene and environment in mental disorders as well as the role of several transmitters/neuropeptides and the different therapeutic strategies. El-Hage et al. (2013) elegantly expose the potential predictors of response/non-response to antidepressants and discuss their clinical and practical implications. Alongside with reviewing several markers that can be used to predict response to pharmacotherapy, they also describe factors that might affect the expression of these markers, including environmental or genetic factors and comorbidities. Then, focusing mainly on the impact of polymorphisms on anxiety-like and depression-like behavior in rodents, Armario and Nadal (2013) discuss how individual differences can contribute to explain differential susceptibility to develop behavioral alterations. They also emphasize methodological problems that can lead to inappropriate or over-simplistic interpretations. Olivier et al. (2013) review the role of the GABAA receptor and the serotonergic system in drug discovery for anxiety disorders. They elegantly highlight how genetic studies aiming to unravel the neurobiology of anxiety have proven to be challenging, and describe how the development of animal models (including genetically modified rodents) has helped to clarify the complex interplay between genes and environment in anxiety-like behaviors. In his opinion article, Dr. Pinna (2014) illustrates the therapeutic strategies to increase neurosteroidogenesis and improve posttraumatic stress disorder by enhancing GABAergic neurotransmission. He also discusses the several therapeutic advantages of targeting allopregnanolone biosynthesis with selective neurosteroidogenic agents. Browne and Lucki (2013) examine the preclinical literature on the antidepressant-like effects of ketamine. After extensively reviewing animal studies which suggest that acute ketamine produces antidepressant-like effect on many behavioral tests, they discuss the potential molecular mechanisms involved. Focusing on direct evidence in the human post-mortem brain as well as rodent genetic and pharmacological studies, Lin and Sibille (2013) summarize the current literature on deficits in somatostatin in neuropsychiatric and neurodegenerative disorders. They conclude that clarifying the role of somatostatin and its regulation of GABA inhibition could provide new therapeutic strategies. Smith et al. (2014) review recent preclinical data on relaxin-3 a newly discovered neuropeptide that binds, and activates the G-protein coupled receptor, RXFP3. They comment on data which suggests that endogenous relaxin-3/RXFP3 signaling promotes arousal and contributes to the central response to stress. This could be relevant and/or potentially translatable to the etiology and treatment of major depression and anxiety. Schirmbeck and Zink (2013) review the contributions of pharmacological and genetic factors in schizophrenia patients with comorbid obsessive-compulsive symptoms (OCS). In this article, they present an in-depth and very detailed coverage of the concepts explaining the co-occurrence of OCS in schizophrenia. They highlight that the effects of environmental factors on onset or symptom has been scarcely investigated and suggest that besides pharmacological treatment as a relevant factor, further environmental factors and gene polymorphisms could play an important role in the development of OCS in schizophrenia. Sumiyoshi's (2013) article aims to provide theoretical issues on atypical antipsychotic drugs in relation to efficacy for treating psychotic symptoms and cognition, as well as safety and tolerability. Based on the fact that no treatments have yet been approved for treating cognitive or negative symptoms in schizophrenia, the author presents a hypothesis for future directions of therapeutics. In that regard, Adams et al. (2013) report that rats with 5,7-DHT-lesions targeting the dorsal hippocampus show potentiated locomotor hyperactivity following treatment with phencyclidine. Given the prominent role of the dorsal hippocampus in spatial information processing, these findings have implications for studies utilizing NMDA receptor antagonists in modeling glutamatergic dysfunction in schizophrenia. Finally, using the tail suspension test, Mitchell et al. (2013) show for the first time that it is possible to detect antidepressant-like activity of drugs in mice as young as P21. Their results suggest that juvenile mice (P21) are less responsive to the antidepressant-like effects of escitalopram than adolescent (P28).

The last five articles cover neuroscience research on drug of abuse. In order to better understand the processes by which peer influences take effect in prairie voles, Anacker and Ryabinin (2013) measure alcohol intake during periods of isolation, pair housing of high and low drinkers, and subsequent isolation. By using a new method (“lickometer” apparatus) and cross-correlation analyses, they managed to differentiate sub-populations of high drinkers that were and were not responsive to social influence to decrease ethanol intake. In another study, Al-Hasani et al. (2013) investigate the interactions between various types of stress paradigms and how they influence kappa opioid receptor (KOR)-dependent reinstatement of cocaine and nicotine preference. They report that chronic mild stress prior to reinstatement prevents a KOR-induced reinstatement response, while acute exposure to stress induces potentiation of KOR-reinstatement. These findings identify KOR as a potentially novel therapeutic target system in drug relapse, anxiety, and depression. Assessing hypothalamic-pituitary-adrenal (HPA) axis activity during withdrawal from chronic ethanol, Pang et al. (2013) found that mice undergoing 2 weeks of alcohol abstinence had significantly greater corticosterone and ACTH levels following a DEX-CRH challenge compared to water controls. Interestingly, environmental enrichment was able to prevent the development of abstinence-associated depression-related behaviors and correct the pathological DEX-CRH corticosterone response. These findings suggest potential for non-pharmacological interventions in the treatment of addiction and depression. Bernheim et al. (2013) summarize the biological factors relevant to adolescent driving risks. The authors discuss the clinical observations in the light of preclinical findings linking impulsivity and emotional reactivity to initiation of drug use and risks of abuse. They conclude that rather than naive, immature and vulnerable, the adolescent brain, particularly the prefrontal cortex, should be considered as prewired for expecting novel experiences. Finally, highlighting the importance of differentiating dopamine D3 from D2 receptors, Le Foll et al. (2014) review the recent methods for measuring D3 receptor occupancy *in vivo*. They present novel methods using [^11^C]-(+)-PHNO and PET which could provide insights into the function of D3 receptors in addiction.

In summary, these studies illustrate how mental disorders can arise from multiple sources. It even seems that the entire body can impact on mental state and psychiatric health (Renoir et al., 2013). We believe that this Frontier Research Topic will stimulate the development of future collaborative and interdisciplinary research.

## Conflict of interest statement

The author declares that the research was conducted in the absence of any commercial or financial relationships that could be construed as a potential conflict of interest.
